# Use of Mobile Application for Improving Drug Compliance and Clinical Outcomes: A Randomized Controlled Trial

**DOI:** 10.1155/ijta/9857793

**Published:** 2026-02-27

**Authors:** Pramod Kumar Mehta, Abhijith R. Rao, Niti Upadhyay, Saad Mustafa, Vasu Digra, Arundhati Sekhar Wariar, Nidhi Soni, Prasun Chatterjee, Avinash Chakrawarty

**Affiliations:** ^1^ Department of Geriatric Medicine, All India Institute of Medical Sciences, Delhi, New Delhi, India, aiims.edu; ^2^ Department of Oral and Maxillofacial Surgery, Sri Sai College of Dental Surgery, Vikarabad, Telangana, India, sscds.edu.in; ^3^ Department of Geriatric Medicine, All India Institute of Medical Sciences, Rishikesh, Uttarakhand, India, aiims.edu; ^4^ Department of Medicine for Older People, Kettering General Hospital NHS Foundation Trust, Kettering, UK

**Keywords:** intervention, KYM, medication adherence, mHealth, multimorbidity, patient satisfaction

## Abstract

**Background:**

Older adults are prone to multimorbidity and polypharmacy, which often lead to adverse outcomes such as increased hospital admissions and treatment nonadherence. Smartphone and internet use among older adults in India is rising, but its potential for addressing healthcare needs like multimorbidity management and drug adherence remains underexplored. The “Know Your Meds (KYM)” Creda Health mobile application (app) on the Google Play Store serves as a digital health assistant, offering features such as medication information, drug interaction insights, and pill reminders to improve health outcomes. This randomized controlled trial is aimed at assessing the effectiveness of the AI‐based mobile app KYM in improving clinical outcomes, medication adherence, and patient satisfaction among older Indian adults.

**Methodology:**

In this randomized controlled trial, 360 participants with multimorbidity (aged > 60 years) were randomly allocated into intervention (*n* = 182) and control (*n* = 175) groups with the intervention group using the KYM app for 12 weeks, whereas the control group received standard conventional healthcare.

**Results:**

Although clinical outcomes like change in blood pressure, HbA1c, and lipid levels did not show a significant difference between the two groups, there was a significant difference in medication adherence at 12‐week follow‐up. However, no significant change was observed in patient satisfaction.

**Conclusion:**

The study highlights the potential of mobile health apps in promoting adherence, though further research is required to evaluate their impact on clinical outcomes with more tailored interventions.

## 1. Introduction

Multimorbidity and polypharmacy are the two major challenges in the management of older adults. The prevalence of multimorbidity can range from 4.8% to as high as 93.1% among these patients, and the prevalence increases with increasing age [1, 2]. Polypharmacy is frequently associated with multimorbidity, and its prevalence among older adults can be as high as 96.5% [3]. Multimorbidity and polypharmacy have been found to result in multiple adverse clinical outcomes, such as falls, frailty, increased hospital admissions, increased duration of hospital stay, and overall mortality [3–5]. In addition, studies have shown that multimorbidity and polypharmacy affect adherence to treatment and quality of life [6–9].

Technology and artificial intelligence (AI) can potentially revolutionize the field of geriatric health by addressing various challenges associated with aging populations. AI holds the potential to deliver advancements in the realms of prevention, diagnosis, and treatment within the healthcare sector. The role of technology and AI is being explored in different aspects of elderly health, like monitoring vitals and falls in older adults living alone, monitoring chronic diseases, assistive devices and rehabilitation, health promotion, and mental health interventions [10, 11].

Technological interventions may also help to improve drug adherence among older adults with multimorbidity. Mobile phone applications that provide drug information and medication reminders are some of the interventions found effective in improving drug adherence [12]. Mobile health (mHealth) apps improve patient involvement and treatment plan adherence with features like virtual consultations and prescription reminders. In addition, with the use of these mobile apps, users can keep an eye on important health indicators, make appointments, and get individualized medical information at their fingertips. They also make it possible for healthcare professionals to monitor patients remotely and collect real‐time data for better decision‐making [13]. The role of mobile apps in improving patient satisfaction and drug adherence has been evaluated in different clinical settings previously [14, 15].

### 1.1. Need for Study

The use of smartphones and the internet is rising among the older population in India. However, its application to address the healthcare needs of older adults, like multimorbidity management and drug adherence, is largely unexplored. The use of AI‐based mobile apps has not been assessed previously in older Indian adults. The “Know Your Meds (KYM)” mobile app is currently accessible on the Google Play Store. KYM serves as a digital health assistant and includes features to improve medication adherence and tracking of health parameters.

In this randomized controlled trial (RCT), we assessed the use of an AI‐based mobile app in improving clinical outcomes and patient satisfaction among older Indian adults with multimorbidity. In addition, we also assessed the difference in medication adherence between the two groups at the end of 12 weeks.

## 2. Methodology

### 2.1. Trial Oversight

The study was a single‐center, prospective, open‐label, RCT conducted at a tertiary care hospital in North India. The trial was designed and conducted by the principal investigator, coinvestigators, and a biostatistician who also analyzed the data. Data collection and management were done by research staff members who were unaware of the trial group assignment.

The trial was funded by Creda Health Know Your Meds India Private Limited. The study protocol was reviewed and approved by the ethics committee of the All India Institute of Medical Sciences, New Delhi, India (IEC‐200/09.04.2021, RP‐15/2021), and registered with the Clinical Trial Registry of India (CTRI/2021/06/034358) prior to its commencement. Participants were recruited after obtaining informed written consent.

### 2.2. Participants

The participants were recruited from the geriatric medicine outpatient department (OPD) of the institute. Adults above 60 years of age with more than one comorbidity and using a smartphone (iOS or Android operating systems) were included in the study after obtaining informed consent. Bed‐bound and critically ill patients were excluded.

### 2.3. Trial Design

#### 2.3.1. Randomization and Masking

**Figure 1:**
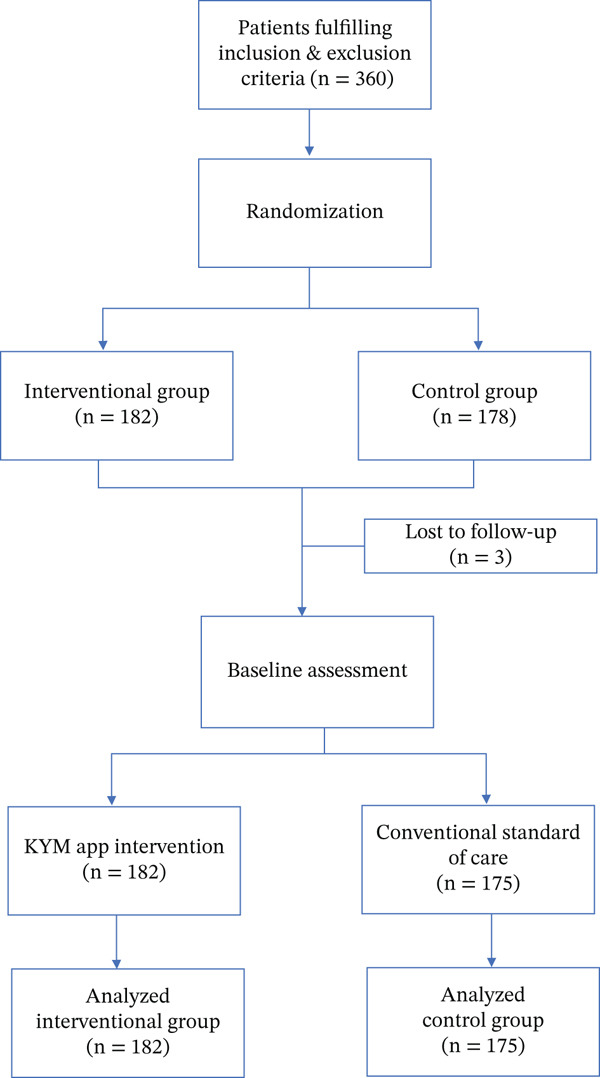
Patient flow diagram: Randomization and assessment.

Participants were randomly assigned in a 1:1 ratio to either the intervention group or the control group (Figure 1). Randomization was carried out with unique identity sequence generation and allocation concealment. A standard randomization procedure for allocation to each intervention stratum was generated using nQuery software Version 2.0 (Statistical Solutions Ltd.). Allocation of the participants to the intervention group was performed using sequentially numbered, opaque, sealed envelopes. The group assignment of the participants was unknown to the research staff responsible for assessing the outcome and performing the final data analysis.

### 2.4. Procedure

The baseline characteristics of all the participants were noted. The variables included age, sex, and educational status (based on the Indian Standard Classification of Education: InSCED 2014). In addition, the patient′s comorbidities, vitals (blood pressure), and biochemical parameters (blood sugar, liver function test, kidney function test, complete blood count, lipid profile, and HbA1c) were recorded.

The patients were then provided with the intervention or standard conventional healthcare for a period of 12 weeks, according to their randomization status. Although there is no standardized duration of follow‐up, we selected a duration of 12 weeks as it provides a fairly long period to assess mobile app usage, adherence, and clinical outcome (e.g. HbA1c). It is in line with previous studies on treatment adherence and takes into consideration feasibility and retention. In a recent systematic review and meta‐analysis on the use of mobile apps and medication adherence, the follow‐up duration in the selected studies ranges from 4 weeks to 12 months (one study), with 12 weeks or 3 months being the most common follow‐up periods (six studies) [16].

### 2.5. Intervention

The KYM mobile app was downloaded from the Google Play Store (https://play.google.com/store/apps/details?id=com.knowyourmeds) on a smart device of the subjects in the intervention group (after informed consent). KYM app serves as a digital health assistant by harnessing the power of AI to deliver personalized information, guidance, and instructions to individuals managing chronic conditions. The application includes features like detailed medication information, insights into potential drug interactions, reminders for pill schedules, recommendations for tracking vital signs, and guidance on doctor‐recommended items such as vaccines and screening tests.

A project staff, separate from those assessing outcomes and performing final analysis, interacted with each patient assigned to the intervention group and assisted them in downloading the app onto the patient′s personal smartphone. Project staff provided an orientation to the patients and their caregivers about the app′s functions, including listing medications and dosages. Patients were given instructions to utilize the app for a duration of 12 weeks. Any change in medications or dosage was updated by the patient or their caregiver. The project staff assisted them if any help was needed in using the app during the study period. The app encompassed the following features:

• Personalized medication dosages schedule with an optional reminder system that could accommodate various treatment regimens
• Medication dosage schedule
• Medication adherence
• Medication reminders

### 2.6. Control: Standard Conventional Healthcare

Prescriptions from patients′ OPD cards were recorded, and the standard of care was provided in accordance with the hospital protocol. These patients received routine care from their clinicians as per established practice. Physicians′ advice was extracted and analyzed from hospital records, with OPD prescriptions documented for record‐keeping and subsequent follow‐up for over a 12‐week period.

In both groups, patients were given diet counseling, medication counseling, and lifestyle counseling, consistent with the practices established before.

### 2.7. Outcome

Outcomes were assessed using the following parameters:

• Vital signs (includes blood pressure)
• Biochemical parameters to look for treatment responses (includes blood sugar, liver function test, kidney function test, complete blood count, lipid profile, and HbA1c)
• Medication adherence was recorded using the Medication Adherence Rating Scale (MARS) having 10 items [17].
• A Patient Satisfaction Questionnaire Short Form (PSQ 18) was administered, and their responses were recorded [18].

### 2.8. Statistical Analysis

No a priori sample size calculation was done. All analyses were performed using STATA Version 14 (*StataCorp. 2015. Stata Statistical Software: Release 14. College Station, Texas: StataCorp LP.*). Normality was tested using the Shapiro–Wilk test. Categorical variables were reported as absolute numbers and percentages, whereas continuous variables were reported as mean with standard deviation (normally distributed data) or median with interquartile range (nonnormally distributed data). To test the association between two groups at baseline, chi‐square test was used for categorical variables, and the independent *t*‐test was used for continuous variables. Differences or changes in outcome variables from baseline were calculated, and the association between the two arms was tested using the Mann–Whitney *U* Test. A *p* value of less than 5% was considered statistically significant.

## 3. Results

A total of 360 patients were randomized (182 in the intervention and 178 in the control group). Three patients did not undergo baseline intervention (all in the control group) and were excluded from the final analysis. The baseline characteristics of the two groups are presented in Table 1. The mean age of the population was 68.88 years (SD 5.25 years). The control arm had an increased proportion of subjects with higher education compared with the intervention arm (*p* value < 0.001). Apart from educational status, the two randomized arms were comparable in terms of other parameters (comorbidities and clinical parameters) at baseline.

**Table 1:** Baseline profile—Intervention group (Arm 1) versus control group (Arm 2).

Variable	Arm 1 (*n* = 182)	Arm 2 (*n* = 175)	*p*
Age (in years) mean ± SD	69.06 ± 5.45	68.69 ± 5.04	0.514
Sex			0.555
● Male: *n* (%)	90 (49.45)	92 (52.57)	
● Female: *n* (%)	92 (50.55)	83 (47.43)	
Education			< 0.001
● 0	80 (44.69)	35 (20.23)	
● 1	23 (12.85)	25 (14.45)	
● 2	24 (13.41)	33 (19.08)	
● 3	11 (6.15)	21 (12.14)	
● 4	26 (14.53)	36 (20.81)	
● 5	15 (8.38)	23 (13.29)	
Comorbidities: *n* (%)			
Hypertension	134 (73.63)	129 (73.71)	0.985
Type 2 diabetes mellitus	86 (47.25)	87 (49.71)	0.642
CLD	2 (1.10)	3 (1.71)	0.680
CKD	12 (6.59)	8 (4.57)	0.406
CCF	4 (2.20)	3 (1.71)	0.742
CAD	28 (15.38)	23 (13.14)	0.545
CVA	7 (3.85)	15 (8.57)	0.063
AF	1 (0.55)	1 (0.57)	0.978
Dyslipidemia	20 (10.99)	13 (7.43)	0.246
Hypothyroidism	30 (16.48)	38 (21.71)	0.208
Post‐COVID	5 (2.75)	4 (2.29)	0.781
DJD	69 (37.91)	69 (39.43)	0.769
Mood disorder	5 (2.75)	7 (4.00)	0.511
Anemia	17 (9.34)	17 (9.71)	0.904
Malignancy	1 (0.55)	5 (2.86)	0.115
Parkinson′s disease	1 (0.55)	1 (0.57)	1.000
Osteoporosis	6 (3.30)	14 (8.00)	0.053
Clinical parameters (mean ± SD)			
Systolic blood pressure (mm Hg)	142 ± 22.32	140.93 ± 21.36	0.513
Diastolic blood pressure (mm Hg)	82.23 ± 10.59	79.98 ± 12.61	0.070
HbA1c (%)	6.56 ± 1.28	6.63 ± 1.28	0.622
Hemoglobin (mg/dL)	12.41 ± 1.73	12.55 ± 1.70	0.457
Serum albumin (mg/dL)	4.48 ± 0.47	4.48 ± 0.39	0.947
Serum creatinine (mg/dL)	1.01 ± 0.55	1.01 ± 0.46	0.956
Total cholesterol (mg/dL)	167.75 ± 43.54	160.73 ± 37.59	0.118
HDL cholesterol (mg/dL)	46.41 ± 11.67	46.29 ± 10.02	0.926
LDL cholesterol (mg/dL)	93.72 ± 37.71	87.46 ± 34.70	0.124
LDL/HDL	2.10 ± 0.87	1.93 ± 0.75	0.072

*Note:* Baseline profile. Chi‐square test and independent *t*‐test were done. *p* value of < 0.05 is statistically significant. Zero, illiterate‐primary education;1, middle education; 2, high school; 3, intermediate/diploma; 4, graduate; and 5, professional/higher education.

Abbreviations: AF, atrial fibrillation; CAD, coronary artery disease; CCF, congestive cardiac failure; CKD, chronic kidney disease; CLD, chronic liver disease; DJD, degenerative joint disease; HDL, high‐density lipoprotein; LDL, low‐density lipoprotein.

The changes in clinical parameters were compared between the intervention and control groups at 12 weeks of follow‐up. (Tables 2 and 3) There was a marginal difference between the two groups in terms of change in systolic blood pressure (SBP), HbA1c, albumin, hemoglobin, and total cholesterol levels. The difference, however, was not statistically significant. When the parameters were assessed in a specific diseased population (i.e., SBP in hypertensives and HbA1c in diabetic patients), the results were similar, with no significant difference observed between the two groups.

**Table 2:** Primary outcome: Change in clinical variables at follow‐up.

Variables	Arm 1 (*n* = 182)	Arm 2 (*n* = 175)	*p*
Systolic blood pressure (mm Hg)	−1 (−9,4)	−3 (−9,5)	0.8345
HbA1c (%)	−0.21 (−0.50, 0.01)	−0.30 (−0.64, 0.07)	0.4431
Hemoglobin (mg/dL)	0.20 (−0.20, 0.60)	0.20 (−0.20, 0.60)	0.5505
Total cholesterol (mg/dL)	−6 (−17, 4)	−4 (−13, 6)	0.1763
HDL cholesterol (mg/dL)	5 (1,10)	5 (0,9)	0.5396
LDL cholesterol (mg/dL)	1 (−10.40, 12.80)	1.50 (−8.65, 10.85)	0.8946
Serum albumin (mg/dL)	0.20 ± 0.49	0.31 ± 0.45	0.0976
Serum creatinine(mg/dL)	0 (−0.10, 0.10)	0 (−0.10, 0.10)	0.4402

*Note:* Mann–Whitney *U* Test has been conducted. *p* value of < 0.05. is statistically significant. All values are shown as median (IQR).

**Table 3:** Primary outcome: Change in clinical variables at follow‐up (in specific subgroups).

Variables	Arm 1 (*n* = 182)	Arm 2 (*n* = 175)	*p*
Systolic blood pressure(mm Hg) in hypertensives subgroup	−3 (−12, 3)	−4 (−11.5, 4)	0.515
Change in HbA1c (%) in diabetic subgroup	−0.39 (−0.87, −0.01)	−0.5 (−0.09, −0.12)	0.274
Change in Hemoglobin (mg/dL) in anemic subgroup	0.59 (0.29, 0.90)	0.5 (0.39, 1.09)	0.871
Change in total cholesterol (mg/dL) in hyperlipidemic subgroup	−25 (−40, −8)	−21 (−32, −11)	0.372

*Note:* Mann–Whitney *U* Test has been conducted. *p* value of < 0.05. is statistically significant. All values are shown as median (IQR).

Patient satisfaction assessed using the PSQ‐18 questionnaire was also found to be similar in the two groups (2.92 ± 0.13 in intervention vs. 2.92 ± 0.11 in the control group, *p* value 0.853). Patient adherence was, however, found to improve in the intervention group compared with the control group (7.09 ± 2.32 vs. 7.61 ± 1.24, *p* value 0.010) (Table 4).

**Table 4:** Secondary outcome.

Variables	Arm 1 (*n* = 182)	Arm 2 (*n* = 175)	*p*
Change in PSQ‐18 score	2.92 ± 0.13	2.92 ± 0.11	0.853
MARS score	7.09 ± 2.32	7.61 ± 1.24	0.010

*Note:* Mann–Whitney *U* Test has been conducted. *p* value of < 0.05. is statistically significant. Data represented as mean ± SD.

Abbreviations: PSQ‐18, The Patient Satisfaction Questionnaire Short Form; MARS, The Medication Adherence Report Scale.

## 4. Discussion

In this RCT evaluating the use of smart app prescription among older adults, no significant difference was found in the clinical outcomes of the two groups. Among the secondary outcomes, the use of a smart app was associated with higher medication adherence but did not significantly change their perceived satisfaction level.

This study evaluated the change in clinical parameters like BP, HbA1c, lipid, hemoglobin, and albumin levels. It was seen that there was a marginal difference in BP, HbA1c, and lipid levels in the intervention and control groups at the end of 12 weeks; however, the difference was not significant clinically or statistically.

Previous studies evaluating the use of mHealth apps in improving clinical and laboratory parameters have shown heterogeneous results on the beneficial effects of mHealth app use. Similar findings were also demonstrated in the technological surrogate nursing RCT, where improvements in self‐care behaviors did not translate into significant between‐group differences in key clinical indicators [19] A RCT conducted by Sun et al. showed clinically significant improvements in blood sugar control among older diabetic patients [20]. Previous RCTs among patients at risk of cardiovascular diseases (CVDs) showed a significant improvement in lipid levels and blood pressure control in the intervention group [21, 22]. Similar results were demonstrated in several systematic reviews and meta‐analyses showing improvement in various CVD risk factors with the use of mHealth apps [23–25]. These effects were a result of improved drug adherence and improved lifestyle changes with the use of mobile apps.

Studies have also shown the beneficial effect of mobile apps in the management of anemia and other nutritional parameters. Such studies are, however, in different clinical settings like sickle cell disease in adults and those with chronic kidney disease‐associated anemia [26, 27].

However, few studies have also shown that there may not be a significant improvement with mHealth apps. Recent RCTs did not find a significant improvement in blood sugar levels among diabetic patients using the mHealth app [28, 29] Furthermore, a couple of studies showed that the changes were significant at initial follow‐ups but declined after the initial 3 months [28, 30]. Factors like a decline in motivation with time and low overall usage of the mobile app were associated with these findings. These studies also emphasized the use of personalized interventions via mobile app, which could improve the patient outcome as each patient has specific needs.

We assessed the patients′ self‐reported satisfaction and adherence to treatment. Although the patient satisfaction scores were similar among the two groups, the intervention group showed improved adherence to treatment. Similar results have been seen in previous studies, where the use of mHealth apps improves adherence to treatment and lifestyle changes in different clinical settings like hypertensive and CVD patients [31, 32]. These studies also showed an improvement in clinical outcomes resulting from improved treatment adherence. However, similar effects on clinical outcomes were not found in our study. Previous studies evaluating the effect of mobile apps on the improvement of patient satisfaction show different results in different clinical settings. An RCT among cancer patients using app‐controlled treatment monitoring and support showed improved patient satisfaction [33]. However, among rheumatoid arthritis patients, the use of the mHealth app did not show any significant improvement in patient satisfaction [34].

## 5. Strengths and Limitations

This study is one of the few RCTs evaluating the use of smartphone‐based mHealth apps among older Indian adults. Older adults with a low‐average literacy rate and higher aversion to using smartphones may not show a significant benefit as has been seen in younger adults. This pragmatic study measured multiple comprehensive outcomes, including multiple clinical and laboratory parameters, as well as an assessment of patient satisfaction and treatment adherence. A fairly long follow‐up period (12 weeks) provided sufficient duration to assess significant changes.

There were also some limitations to this study. The study was conducted in a major hospital in Delhi, which is an urban setting, and hence, the population may not be representative of the general population of India. Educational status is an important factor determining the use of mobile phones and mHealth apps. The difference in the educational status between the two groups could affect the study outcomes and is another significant limitation of the study, and baseline study was not assessed. The mHealth app focused on pill reminders, lifestyle recommendations, and tracking of vitals and blood investigations. However, the mobile app was not developed to target any particular disease or condition. Similarly, the population was not limited to any particular comorbidity. As observed above, targeted and individualized interventions show significant improvement on the outcome assessed.

## 6. Conclusion

This pragmatic RCT evaluating the effect of the mHealth app on clinical and laboratory outcomes among older Indian adults did not show any significant improvement. However, there was good acceptance of the mobile app and a significant difference was seen in treatment adherence, even though no significant effect was seen on patients′ self‐reported health satisfaction. However, it is too early to dismiss the use of mHealth apps among older Indian adults. mHealth applications targeting specific diseases and individualized intervention may show improvement in clinical outcomes.

## Funding

This study was supported by Know Your Meds India Private Limited.

## Conflicts of Interest

The authors declare no conflicts of interest.

## Data Availability

The data that support the findings of this study are available from the corresponding author upon reasonable request.
